# The Impact of Maternal Interpersonal Violent Trauma and Related Psychopathology on Child Outcomes and Intergenerational Transmission

**DOI:** 10.1007/s11920-024-01491-7

**Published:** 2024-03-01

**Authors:** Shannen Graf, Daniel S. Schechter

**Affiliations:** 1 Division of Child and Adolescent Psychiatry, Department of Psychiatry, Lausanne University Hospital (CHUV), SUPEA–Unité de recherche, Avenue d’Echallens 9, 1004 Lausanne, Switzerland; 2 Department of Psychiatry, Faculty of Biology and Medicine, University of Lausanne, Lausanne, Switzerland; 3 Department of Child and Adolescent Psychiatry, New York University Grossman School of Medicine, New York, NY USA

**Keywords:** Maternal trauma, Posttraumatic stress disorder, Child outcomes, Interpersonal violence, Intergenerational transmission of trauma

## Abstract

**Purpose of Review:**

This review aims to outline some consequences that maternal history of trauma with and without related psychopathology, such as posttraumatic stress symptoms (PTSS), can have on their children’s development and functioning. It then addresses mechanisms through which intergenerational transmission of interpersonal violence (IPV) and related psychopathology may occur.

**Recent Findings:**

Findings include the effects of maternal IPV experience and related psychopathology on child social-emotional and biologically-based outcomes. This includes increased developmental disturbances and child psychopathology, as well as physiological factors. Secondly, the review focuses on psychobiological mechanisms by which maternal experience of IPV and related psychopathology likely trigger intergenerational effects.

**Summary:**

Maternal IPV and related psychopathology can have a negative impact on several areas of their child’s life including development, interactive behavior, psychopathology, and physiology. This transmission may partially be due to fetal and perinatal processes, genetic and epigenetic effects, and interactions with their parents.

## Introduction

Traumatic life events are those that are overwhelmingly shocking and/or distressing and that have a negative if not potentially damaging impact on the organism psychologically and even, in many instances, physically. Current thinking is leaning towards replacing the notion of “traumatic life events” with “traumatic experiences” in order to take into account the subjective impact and meaning of the event to a given individual. The event, by the Diagnostic and Statistical Manual of Mental Disorders, Fifth Edition (DSM-5) definition, involves “actual or threatened death, serious injury, or sexual violence” [1, 2]. It is estimated that as many as 55.8 to 85.4% of individuals will experience at least one such traumatic event in their lifetime [3, 4]. Even being a witness to such events or learning about their occurrence, particularly to a family member or close friend, can also have deleterious effects and thus qualify as a traumatic experience. For example, in the case of a child witnessing domestic violence, there is an increased risk for that child to develop PTSS or even, for a certain number, go on to perpetrate domestic violence during adulthood [5]. The notion that primary caregivers can be mortally endangered or endangering is indeed an existential threat to the infant who is completely dependent on their caregivers for nurturance, protection, and survival. In sum, the majority of the population will experience traumatic life events, either directly or indirectly, with a significant proportion suffering a variety of sequelae.

Interpersonal violence (IPV) related events are considered to be among the most “traumatogenic” types of life events [6]. By IPV, we refer to physical and/or sexual assault and/or abuse across the lifetime, which can take the form of experiencing or witnessing, particularly in the case of children, domestic violence. The earlier, more repeated, and more injurious the violence, the greater the traumatic impact on the individual is going to be, resulting in an increased likelihood of maladaptive outcomes, most notably acute and posttraumatic stress symptoms (PTSS) which can develop into posttraumatic stress disorder (PTSD) [7–9]. The presentation of PTSD includes reexperiencing symptoms (i.e., flashbacks, nightmares), avoidance of any reminder of the event, hypervigilance, and negative cognitions (i.e., guilt, expectations of catastrophe). PTSD has a lifetime prevalence of around 3.9% in the total population, and 5.6% in the population who claim to have experienced a traumatic incident or a series of events [10]. PTSD is an impairing condition for most of those who suffer from it [11]. Exposure to multiple or repeated adverse childhood events (ACEs) that encompass IPV from infancy and early childhood is frequently associated with complex or developmental PTSD (cPTSD), as well as increased risk for a range of mental and physical health problems from cardiovascular disease to cancer [12]. cPTSD is a form of PTSD that, in addition to the essential PTSD criteria noted in the DSM-5-TR, also includes a disruption in self-regulatory capacities, such as emotion regulation and interpersonal relationships to cite a few [13]. The prevalence of PTSD and particularly cPTSD is common among adults with histories of multiple ACEs, especially those involving a primary attachment figure and dating back to early sensitive periods in development. Of note, adults with multiple ACEs very often go on to experience subsequent assaultive trauma at rates ranging from 30% in a community sample to 50% in a public mental health facility [14, 15].

More than the consequences to the people affected, experiencing IPV and/or developing PTSS can also have consequences to those individuals’ loved ones. In particular, the fact that a mother may have experienced IPV or is suffering from PTSS has been shown to have several effects on her relationship with her child, as well as said child’s physical and mental health directly [16••, 17, 18]. This is one aspect of the phenomenon known as the intergenerational transmission of trauma, which refers to the fact that some sequelae of traumatic events experienced by caregivers, such as suffering from PTSS, may negatively impact their children’s development and functioning [19]. The consequences of this transmission are very diverse, and there is an urge to study them scrupulously as well as how this transmission occurs in order to interrupt it.

Thus, this review has as its objective to examine the research that pertains to the intergenerational transmission of the effects of maternal IPV, particularly that of related PTSS to the child, and how this transmission from mother to child occurs. What effect do these factors have on the child’s outcomes? Through which mechanisms does this transmission take place? Can it occur even before birth? This review will look at the research over the past 3 years to bring us up to date in answering these questions, looking at different domains from impacts on attachment-related, psychopathological and neurobiological outcomes, to potential transmission mechanisms. In writing this review of the scientific literature over the past 3 years, the authors found the research included particularly timely and important given the rise as high as 5–10% globally in the number of reported cases of domestic violence and child maltreatment since the start of the COVID-19 pandemic [20, 21].

## Impacts of Maternal Interpersonal Violent Trauma History on Symptomatology on Children

The consequences of maternal IPV (e.g., childhood physical and/or sexual abuse, exposure to and victimization related to physical and/or sexual violence) are highly varied and take different forms, some of which we will develop in the remainder of this paper.

### Developmental Disturbances

From a developmental point of view, maternal exposure to physical violence within the parental couple was associated with poorer child motor development scores [22, 23]. Results are unclear with respect to maternal childhood exposure to violence: Chung et al.’s [24] study showed no association between maternal childhood exposure to home violence and offspring’s fine motor skills and language development. Interestingly, it seemed that maternal childhood exposure to community violence was, however, associated with reduced offspring’s fine motor skills but better receptive language [24]. Yet it is also possible that some of the mothers who have been exposed to domestic violence during childhood and develop PTSS may behave less sensitively with their child such that the child does not experience sufficient maternal emotional availability, reciprocity, and stimulation to develop their language skills adequately [25]. Maternal IPV history is also consistently linked with diminished executive functioning abilities, such as cognitive flexibility and inhibition control, either directly or indirectly [26•, 27•, 28]. Mothers’ history of physical, sexual, and even psychological or emotional abuse can also have an impact on their children’s regulatory capacities, including negative emotionality, sleep disturbance, and eating problems for example [29•, 30•].

In terms of behavior, one of the effects that a maternal past IPV history can have on their offspring is child withdrawal behavior, consisting of the child engaging in less social approach and interaction [31]. History of maternal IPV has been shown to be indeed associated with social withdrawal during infancy [32•]. PTSS, however, were only associated with infant social withdrawal in the presence of comorbid major depressive disorder, indicating a shared effect [32•]. Both past maternal IPV and current depressive symptoms seem associated with atypical maternal interactive behavior which mediated the effects of the former on infant social withdrawal at 6 months.

Additional studies of mothers with a higher number of ACEs, greater adult domestic violence exposure and related PTSD vs less exposure to IPV and no PTSD had children that showed increased dysregulation of arousal with increased fear-potentiated startle response to a fear and safety learning task and with an attentional bias to displays of angry faces [33, 34••]. These more recent findings echo a number of prior studies of physically maltreated children who show either prominent attention or avoidance to angry faces depending on the study and presence of PTSD, as well as one study of children of mothers suffering from IPV-PTSD [35–38].

### Child Psychopathology

The two broad categories of child symptoms as potential indicators of psychopathology are those of internalizing and externalizing symptoms. Internalizing symptoms are subjectively reported often painful or distressing mental states and/or somatic sensations that may or may not show outward expression. This category includes, for example, anxiety and depression, as well as somatization symptoms. The literature reports that children and adolescents of mothers having experienced adverse events and/or suffering from PTSS have higher levels of anxiety and depression [39••, 40, 41]. However, some studies found an association between maternal IPV history and increased child somatization, and others did not [39••, 42]. A possible explanation could be that this link is moderated by other factors: Glaus et al. [43] showed an association between the two that was mediated by maternal somatization, providing a potential mediator in this relationship that could explain the discrepancies between studies. Yet in any case, the greater the maternal experience of adverse events and suffering from PTSS, the greater the severity of internalizing symptoms in the offspring [16••].

With respect to externalizing symptoms, which refer to symptoms that are in the form of observable behavior, such as aggression, hyperactivity, impulsive, and disruptive behaviors that are not aggressive, maternal lifetime adverse experiences have been shown to enhance them, whether directly or indirectly [40, 44, 45•]. The more adverse experiences the mother has encountered and the more PTSS from which she suffers, the more externalizing behaviors her child seems to have [46, 47, 48•, 49].

Maternal IPV history from childhood and related psychopathology have also been associated with child and adolescent psychiatric disorders that meet diagnostic criteria according to the International Classification of Diseases (ICD-10/11) or the DSM-5 [50, 51]. First, maternal childhood IPV exposure and emotion dysregulation are robustly associated with increased maternal potential for child abuse. Additionally, they contribute to the occurrence of maternal depressive symptoms and child trauma exposure due to inadequate protection of the child. Both increased maternal potential for child abuse and decreased protection from threats by others have been linked to the development of child PTSD [52•]. Secondly, maternal IPV history and related PTSS are known to be linked to negative affectivity with an increased likelihood of mood disorders in their children [53•, 54••]. A history of maternal childhood IPV is associated with more anxiety in their offspring when they are toddlers, and maternal PTSS are linked to more anxiety in their adolescent children [55, 56]. Maternal childhood IPV exposure has, moreover, been associated with a greater risk for psychotic disorders among offspring who display psychotic symptoms such as hallucinations and delusions, following from a greater number of traumatic events among those offspring [57•].

### Biological and Physiological Impacts

In addition to the psychological impacts, maternal exposure to IPV and related PTSS can also have a large range of physiological impacts on children. These impacts can already be reflected by perinatal indexes. Firstly, infants of mothers with a history of exposure to IPV have more risks of being born via caesarean section and of being born prematurely (i.e., < 37 weeks) compared to infants of mothers who did not experience IPV [58–60]. These children also have lower birth weights and are more likely to be admitted to the neonatal intensive care unit [61, 62].

Results are also inconsistent about the effect on child growth: Apanasewicz et al. [63] showed that children of mothers with multiple ACEs weighed more at 5 months, but this association was not found with 1-year-old children [24]. In general, infants of maltreated mothers have more health concerns, and this persists throughout the years [64, 65]. Infants of mothers with more IPV exposure also showed lower respiratory sinus arrhythmia regulation (i.e., vagal tone), indicating lower levels of physiological regulation with increased reactivity to stressors such as the still-face procedure [66••].

It is also associated with subsequent physiological aspects. For example, children of mothers who have a history of childhood maltreatment are more likely to be asthmatic [67•]. In females only, maternal maltreatment history was also associated with a higher risk for obesity [67•].

Finally, infants of mothers with a greater number of ACEs have been shown to have correspondingly shorter telomere length, a biomarker of cellular stress and aging, across infancy [68••]. This finding was coupled with increased aggressive infant behavior at 18 months [68••].

## Possible Transmission Mechanisms

The mechanisms through which maternal IPV and related PTSS can have an impact on their child have been and are still being thoroughly studied to be able to reduce their transmission to a minimum. From biological to environmental factors, there is a variety of variables that can contribute to this intergenerational transmission.

### Fetal Factors

One way that the effects of IPV can be transmitted already occurs even before the baby’s birth, during fetal development while the mother is pregnant. The placental expression of certain genes is altered in women who have experienced IPV-ACEs and subsequent IPV. One such gene is that which encodes the enzyme O-linked N-acetylglucosaminyltransferase (OGT) that is involved in nutrient sensing. Others include the gene that codes for the glucose transporter GLUT1, playing a role in nutrient transport across the blood–brain barrier, and the gene coding for the hypoxia inducible factor 3 subunit alpha (HIF3A), regulating adaptive responses to low oxygen tension, but only in the case of male fetuses [69••]. Fetal adrenal volume (FAV) is also altered, but results are inconsistent, with some finding lower FAV in male fetuses of mothers with high adversity at 21 weeks gestation but no difference at 29 weeks, while others found a slower growth of FAV between 19- and 28-weeks’ gestation in fetuses of mothers with high adversity regardless of fetus sex [70, 71]. Placental levels of corticotrophin-releasing hormone (pCRH), involved in the hypothalamic–pituitary–adrenal (HPA) axis related to responses to stress, seem to be higher during the third trimester and rise more during pregnancy for women with more ACEs [72•]. In relation to this, the placenta generally blocks most of the passage of maternal cortisol due to the action of a specific enzyme called 11β-Hydroxysteroid dehydrogenase-2 (11beta-HSD-2). However, if a mother experiences acute stress during pregnancy, cortisol may not be sufficiently filtered out and this can affect fetal brain development [73]. Additionally, fetal growth factors are also negatively affected by maternal childhood adversity [74].

### Autoimmune Factors and Perinatal Transmission

At least one study has supported that mothers with persistently elevated depression, anxiety, and PTSS postpartum show an upregulation of genes activated by transcription control pathways associated with inflammation [75]. There is some evidence of prenatal and possibly post-natal (i.e., breastmilk) transmission of inflammatory factors from women suffering from depression or PTSS to their offspring, yet that evidence is inconsistent across studies and requires further research in humans [76, 77]. In rodent models, it has been demonstrated that maternal immune factors can impact fetal brain development, yet not specifically with respect to maternal psychopathology [78]. A further study suggests that research in this area pay particular attention to the possibility of maternal mitochondrial transmission [79].

### Genetics and Epigenetics

Another type of mechanism for IPV and related PTSS transmission may be through genetic and epigenetic material.

For example, in terms of genetics, one study found that maternal PTSD predicted increased child symptoms for children carrying the minor T-allele (CT/TT) but not those homozygous for the major C-allele of the FKBP5 gene [80••]. These findings provided the first evidence that a gene x environment interaction involving FKBP5 and child trauma exposure extended to maternal PTSS.

In terms of epigenetics, infants of mothers with interpersonal violence-related PTSS displayed differential DNA methylation at several points in the promoter regions of specific stress-related genes, which can alter the gene’s expression of proteins, such as for example receptors [81–83]. Notably, there might be alterations in sections involved in the hypothalamic pituitary adrenal axis. Indeed, Cordero et al. [84•] showed in a Swiss sample that the percentage of methylation of the promoter region of the infant glucocorticoid receptor gene NR3C1 was robustly correlated with that of the maternal gene NR3C1 only for the group of mothers suffering from PTSD. They also found that lower methylation in mothers was negatively and significantly correlated to externalizing symptoms in the child at school age per maternal report [84•]. As found in previous studies of adults with cPTSD, lower methylation of mothers’ NR3C1 promoter region was associated with the presence and severity of cPTSD. Of note, another study showed that Cameroonian mothers’ exposure to domestic violence was not significantly associated with lower maternal methylation of the NR3C1 promoter region [85]. However, this study did not take maternal psychopathology, notably PTSS, into consideration. Interestingly, the study showed that greater methylation of the promoter region of the NR3C1 gene among the violence-exposed mothers was significantly associated with increased anxiety symptoms in preschool-age children [85]. These differences indicate that further studies are needed to explain the different outcomes with a larger sample across various ethnicities.

### Interactive Behavior with Traumatized Caregivers During Early Sensitive Periods

Apart from the aforementioned biological factors, behavioral factors can also play a role in the transmission of IPV and related PTSS. After suffering from IPV, including domestic violence, mothers can show different patterns of interactive behavior with their children than mothers who did not experience such violence during their life. For example, mothers who were physically maltreated during childhood or who witnessed domestic violence during the same period are at higher risk of perpetrating child physical abuse themselves [86].

Apart from having an influence on different areas of the mother’s life, maternal exposure to IPV and subsequent PTSS can also have an important effect on the interactions between her and her child, contributing to its transmission. It has been shown that childhood moderate and severe trauma, as well as current stressful events, diminishes maternal emotional availability when interacting with her 6-month-old child [87•]. However, it seems like maternal PTSS enhances responsive parenting from the mother in early interactions with her child [54••, 88].

Child regulatory capacities have been linked both to child biology, the caregiving environment, and their interaction, in particular the security and organization of attachment and related mentalization, which can both be transmission mechanisms [89]. About the former, studies had initially not taken maternal ACEs and subsequent IPV history into account when studying attachment and found that maternal perinatal depression was linked to insecure and disorganized attachment. One study of 224 mothers and toddlers found that, with clear control groups, only maternal ACEs and subsequent domestic violence exposure were associated with insecure and/or disorganized attachment, the latter a potent risk factor for internalizing, externalizing, and regulatory difficulties, showing how disturbed attachment may contribute to the transmission of trauma [90]. Contrary to previous findings, no association was found between attachment quality and perinatal depression alone [90]. Mentalization, for its part, is the ability to infer mental states in self and others and is measured as “reflective functioning.” Maternal history of childhood maltreatment and related psychopathology have been linked to greater child behavioral dysregulation through less parental reflective functioning, along with greater maternal depressive symptoms, coupled with increased potential to maltreat her own children [29•, 91, 92•].

As just mentioned with respect to mentalization, it is important to note that maternal behavior is partially influenced by maternal thoughts and mental representations, which can also be altered in women having experienced a potentially traumatic event. For example, the intensity of maternal childhood maltreatment was associated to the intensity of the disruption of prenatal maternal representations [93•]. This did have an impact on the mother–child relationship, with an association with less secure attachment when the child was 1 year old [93•]. Additionally, their knowledge on parenting prior to the baby’s birth had an impact on child problems later, with more knowledge buffering the impact of maternal childhood exposure to IPV on child outcomes, and with less knowledge increasing it [46].

## Conclusions

In conclusion, this review has shown that mothers’ experience of IPV, particularly when beginning early in life, has multiple negative effects on child development. Its effects can begin even before birth as evidenced by greater risk for premature birth, shortening of child telomeres, and fetal programming. Its effects continue through middle childhood and adolescence with an impact on the development of a range of child psychopathology.

What is striking is that many studies examine only maternal experience of IPV without the evaluation of related psychopathology such as PTSS and depression, which is very often present and which, in multiple studies, is a prerequisite for many of the untoward outcomes. Linked to these alterations in mind, brain, and physiology, there exist multiple pathways resulting in intergenerational transmission of trauma-related psychopathology and violence, as well as associated risk. These pathways include, but are not limited to, genetic polymorphisms and epigenetic modification of stress-related genes. This review additionally encompassed evidence of abnormalities in maternal stress physiology and inflammatory processes that can be transmitted prenatally to the fetus. Similarly, these pathways towards intergenerational transmission have been shown to involve a caregiving environment characterized by insensitive or atypical maternal behavior that can be intrusive, withdrawing, dissociated, with mismatched affect, and/or reversed roles. Maternal avoidance and dissociation can also lead to a mother being inadequately protective, as mentioned, as a result of maltreatment by caregivers other than mother, whereas some highly insensitive, hostile, and intrusive mothers may themselves be prone to maltreat their own children. These alterations in the caregiving environment may at their best represent an adaptation—if one takes an evolutionary perspective—to prepare the next generation for a potentially dangerous environment, and at their worst, represent a maladaptation that places the child at a disadvantage to thrive in a complex social world with sufficient flexibility. In either case, this review has made the case for awareness of risk factors for greater aggressivity and impulsivity in certain children that make them more likely to perpetrate violent behavior as adolescents and young adults, thus perpetrating the cycle of intergenerational violence and related psychopathology. While we are learning more about the consequences of maternal IPV and related psychopathology to the fetus, infant, and early mother–child relationship, further research is needed on how to intervene effectively and early based in part on what has been learned from the reviewed research in recent years, in order to interrupt intergenerational cycles of violence and despair.

This review, as an important limitation, has focused exclusively on mothers’ experiences of IPV and their impact on child outcomes. Why only mothers and not fathers? Mothers are most often the primary attachment figure who spend the most time from conception on with the fetus and then the infant. Yet research needs to incorporate additional attachment figures, the other parent, and in some cases the grandparent or aunt/uncle that is essential to the caregiving environment.

In conclusion, this review of published studies over the past 3 years has demonstrated significant associations between maternal adverse life events in childhood and subsequently — with and without related PTSS having been measured depending on the study — and child psychological, behavioral, and biological outcomes over time. The review goes on to examine the recent literature on possible mechanisms that contribute to the intergenerational transmission of violence, violent trauma, and related psychopathology. Clinical implications of this review include consideration of likely risk factors for intergenerational transmission in an effort to improve the ability to evaluate and intervene early when infants and parents are motivated and sufficiently plastic to sustain enduring change given the sensitive periods implicit to infancy, early childhood, and new parenthood.
